# A Review on the Morphology and Material Properties of the Gas Separation Membrane: Molecular Simulation

**DOI:** 10.3390/membranes12121274

**Published:** 2022-12-15

**Authors:** Yilin Liu, Na Li, Xin Cui, Weichao Yan, Jincai Su, Liwen Jin

**Affiliations:** 1 School of Human Settlements and Civil Engineering, Xi’an Jiaotong University, No. 28 Xianning West Road, Xi’an 710049, China; 2 School of Chemical Engineering and Technology, Xi’an Jiaotong University, No. 28 Xianning West Road, Xi’an 710049, China; 3 School of Life Sciences & Chemical Technology, Ngee Ann Polytechnic, 535 Clementi Road, Singapore 599489, Singapore

**Keywords:** gas separation, composite polymeric membranes, molecular simulation, membrane morphology, transport properties

## Abstract

Gas membrane separation technology is widely applied in different industry processes because of its advantages relating to separation performance and economic efficiency. It is usually difficult and time consuming to determine the suitable membrane materials for specific industrial separation processes through traditional experimental research methods. Molecular simulation is widely used to investigate the microscopic morphology and macroscopic properties of materials, and it guides the improvement of membrane materials. This paper comprehensively reviews the molecular-level exploration of the dominant mechanism and influencing factors of gas membrane-based separation. The thermodynamics and kinetics of polymer membrane synthesis, the molecular interactions among the penetrated gases, the relationships between the membrane properties and the transport characteristics of different gases in the composite membrane are summarized and discussed. The limitations and perspectives of the molecular simulation method in the study of the gas membrane separation process are also presented to rationalize its potential and innovative applications. This review provides a more comprehensive reference for promoting the materials’ design and engineering application of the gas separation membrane.

## 1. Introduction

Unlike the traditional gas separation processes, the novel membrane separation technology utilizes the high-molecular polymer membranes to selectively “filter” the target gas. This technology was industrialized in the late 1970s, because of its flexible and stable operation, small size, economic viability, low energy consumption and good separability [1,2,3]. Membrane technology has been highly developed in the separation of liquid components such as seawater desalination [4], oil-water separation [5] and water treatment [6], etc. The separation characteristics of these membrane processes and their influencing factors have been thoroughly investigated through theory, experiment and simulation. In addition, polymer membranes have been applied in different industry gas processes, such as carbon dioxide recovery [7,8], natural gas sweetening [9,10], oxygen production [11,12], volatile organic compound (VOC) recovery [13,14], air dehumidification [15,16], biogas upgrading [17,18], helium recovery from natural gas [19,20], nitrogen enrichment [21,22] and ammonia synthesis purification gases [23,24]. Under the impetus of concentration, pressure or electric potential difference, the gas separation membrane depends on the different penetration rate of the components in the mixture across the non-porous dense layer to achieve the separation of each component. Generally, the non-porous membrane (namely, the dense membrane) is very thin, and its thickness is in a nanometer scale. It needs to rely on the porous membrane as the support to supply adequate mechanical strength. Therefore, the composite membranes with a porous membrane and dense layer are usually fabricated for the gas separation process.

Some difficulties still limit the large-scale promotion of membrane separation technology in industry, especially, the unsatisfactory permeability and separation performance of the gas separation membrane. The gas permeation through the thin non-porous separation layer coated on a porous ceramic membrane is dominated by the characteristics of separated gas, the membrane morphology and the material of the membrane selective layer [8]. There is a lack of membrane design theory and unclear relationships between the membrane material properties and the membrane morphology evident in the fabrication of membrane materials. In most membrane separation processes, the actual membrane materials used are usually screened in the commercial membranes by experimental methods, and these materials are usually not the most suitable choice for the application system. In addition, the preparation of composite membranes is complicated and mainly lies in the microstructure control of porous membranes and the thin and defect-free selective layer. To determine the quantitative control method of the membrane preparation process, it is necessary to explore the gas transport characteristics, the dominant mechanism and the influencing factors of gas molecules across the dense layer and the porous membrane.

Consequently, this study aims to succinctly summarize the current state-of-the-art on gas separation at a molecular level, and to provide an insight into the morphology and material properties of the gas separation membrane. To reach this goal, first, the transport mechanism of gas molecules is introduced, followed by the correlation between the membrane morphology and the membrane properties. Second, the kinetic properties of gas molecules in the composite membranes are reviewed and analyzed. Finally, the limitations and perspectives of gas separation membrane technology are concluded and discussed to promote the engineering application of the gas separation membrane.

## 2. The Transport Mechanism of Gas Molecules

**Figure 1:**
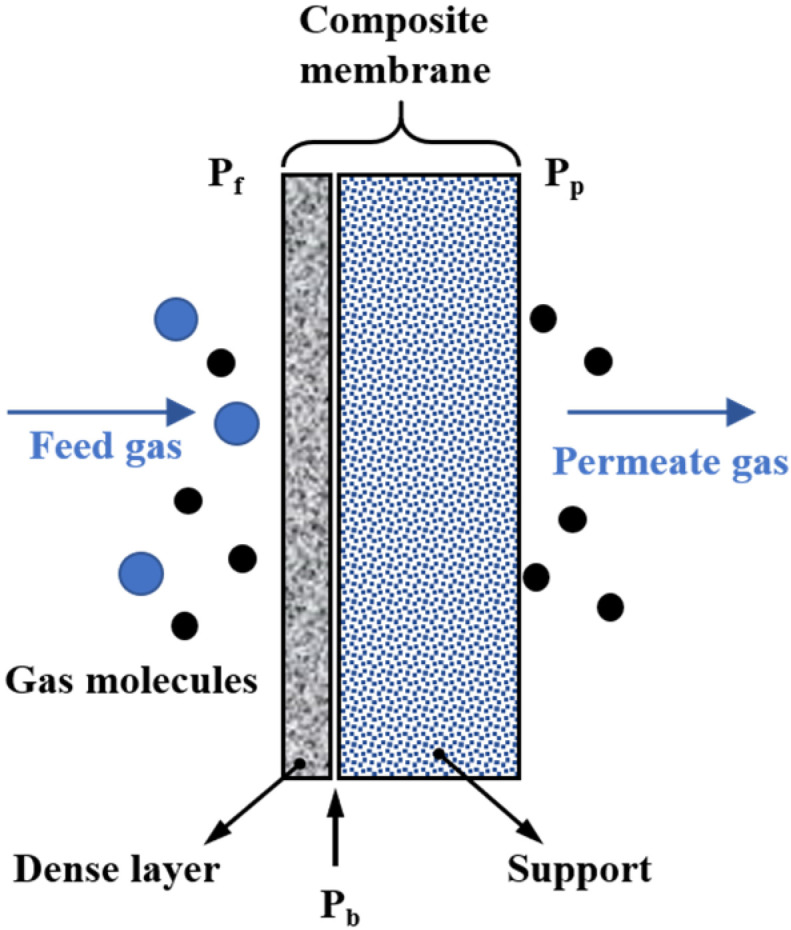
The transport process of gas molecules through a composite membrane.

As demonstrated in Figure 1, a typical structure of the composite membrane for gas separation consists of a porous support membrane and a non-porous selective layer. While the porous membrane (the pore size is up to 10 μm) possesses lower selectivity and a high permeance, the non-porous membrane (the dense separation layer) has good selectivity and weak permeance [25]. The transport mechanism of gas molecules in these two types of membranes is quite different. The porous membranes allow the small molecules to pass through their micro-pores, whereas the dense or nonporous membranes allow the small molecules to be transported through the intermolecular microchannel of the membrane materials.

### 2.1. The Porous Membrane

The diversities in pore distribution and surface characteristics of membrane materials lead to different interactions between gas molecules and membranes resulting in different characteristics of gas transportation in the porous membrane. The flow characteristics of gas in porous materials are determined by a characteristic parameter, the Knudsen number (*Kn*). *Kn* is defined as the ratio of the number of molecular-molecule collisions to the number of molecular-wall collisions. Based on the region of *Kn*, the molecular motions in porous membranes are divided into three types: Knudsen diffusion, surface diffusion and molecular sieving.

(1) Knudsen diffusion.

**Figure 2:**
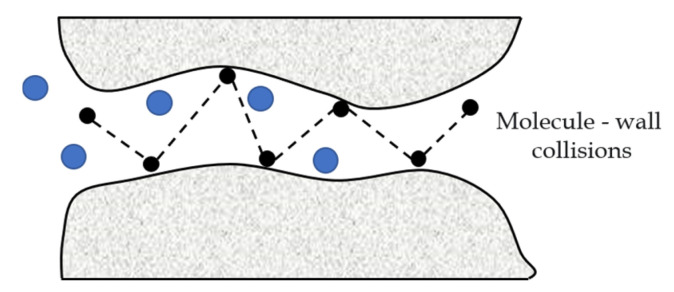
A schematic diagram of the Knudsen diffusion.

When the mean free path of the gas molecule is larger than the micropore diameter, the molecular-wall collisions are much more intense than the molecular-molecule collisions. In this case, the Knudsen diffusion is dominant with the schematic diagram as shown in Figure 2 and the gas flux (*f_k_*, kg/(m^2^ s)) is described as follows [26]:

(1) $$f_{k}=\frac{8r_{p}}{3{\left(2\pi MRT\right)}^{\frac{1}{2}}}$$

where *M* is the gas molecular weight (kg/mol), *r_p_* is the membrane pore radius (m), *T* is temperature (K) and *R* denotes the gas constant (J/mol K).

(2) Surface diffusion.

**Figure 3:**
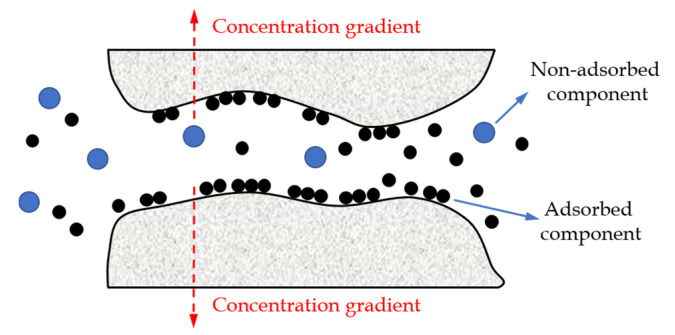
A schematic diagram of the surface diffusion.

Based on the chemical interactions with the membrane surface, gas molecules can be adsorbed on the membrane surface. Figure 3 displays that the molecules adsorbed on the pore wall (black dot) diffuse along the surface under the concentration gradient in the adsorbed state. In this process, the adsorbed component diffuses faster than the non-adsorbed component (blue dot), resulting in a difference in permeability and achieving the separation purpose of a specific component. At low surface concentrations, the surface flow rate of pure gas can be expressed by Fick’s law [27]. The gas flux (*f_s_*, kg/(m^2^ s)) is described as follows:

(2) $$f_{s}=-\rho (1-\varepsilon )\mu_{s}D_{s}\frac{d_{1}}{d_{2}}$$

where *ρ* and *ε* are the density (kg/m^3^) and porosity of porous media, respectively; *μ_s_* is the shape factor; *d*_1_*/d*_2_ is the membrane thickness change due to the surface adsorption; *D_s_* is the surface diffusion coefficient (cm^2^/s).

(3) Molecular sieving.

**Figure 4:**
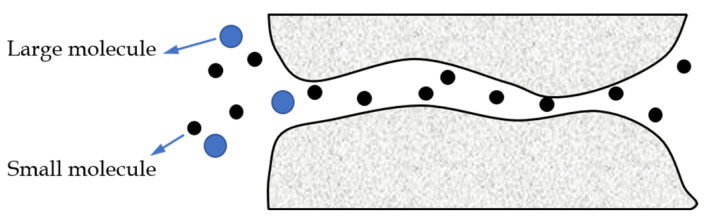
A schematic representation of the molecular sieving effect.

As displayed in Figure 4, when the molecular scale is comparable to the membrane pore size, the membrane surface can be regarded as possessing numerous micropores and the separation based on the difference in the size of the gas molecules can be realized [28]. This is known as the molecular sieving effect, which is a relatively ideal separation method. In order to achieve a low energy consumption and a large and high selectively mixed separation, the thickness of the molecular sieve membrane should be controlled. However, it is still a challenge to fabricate the defect-free microporous membranes with a thickness of less than 20 nm under the existing membrane materials and conditions [29].

Judging the diffusion form of small molecules across the membrane is critical to the study of the gas separation performance of membranes at a molecular level. Knudsen diffusion is the most common form of molecular transport in porous membranes during the gas membrane separation process [30,31,32,33]. The augmentation of temperature generally leads to a larger mean free path of gas molecules, so the temperature of the gas mixture should be sufficiently low. At a given temperature, the Knudsen diffusion rate is positively related to the pressure difference, so the pressure difference that drives the diffusion behavior should be improved as much as possible. The gas with good condensability is easily adsorbed in the pore wall, and in this case the surface diffusion is significant. The smaller pore size and lower operating temperature result in a more significant surface diffusion behavior. For most gases, surface diffusion always accompanies Knudsen diffusion. The adsorption capacity and diffusion flux of gas molecules in the porous membrane are enhanced by expanding the surface area, reducing the pore size and improving the membrane adsorption performance [34]. Under a certain pressure difference, the chemisorption rate increases with the increase in temperature, so the surface diffusion rate is improved. However, the increase in the molecular mean free path with the temperature will cause the decrease in the Knudsen diffusion rate. Under a certain temperature, the surface diffusion rate first increases and shows a saturated trend, further increasing the pressure difference, while the Knudsen diffusion rate is proportional to the pressure difference.

### 2.2. The Non-Porous Membrane

Non-porous membranes (inorganic or polymer materials) possess permeability, and most are resistant to a high temperature, pressure and chemical corrosion. Common non-porous membrane materials mainly include rubber polymers and glass polymers. The solution-diffusion mechanism is dominant in the gas molecules’ permeation in the non-porous membrane. The solution-diffusion model is the most accepted mechanism to explain the molecular transport process in pervaporation, gas permeation, reverse osmosis and dialysis.

The driving energy of gas transport in the non-porous membrane comes from the chemical potential difference caused by the pressure difference [35,36], concentration difference [37,38] or potential difference [39,40] between the two sides of the membrane. The purpose of separation can be achieved according to the difference in the relative transfer rate of the components under the operating conditions. Generally, the permeability, diffusion and solution coefficients depend on the properties of membrane materials, chemical characteristics of gases and the pressure and the temperature of gases.

**Figure 5:**
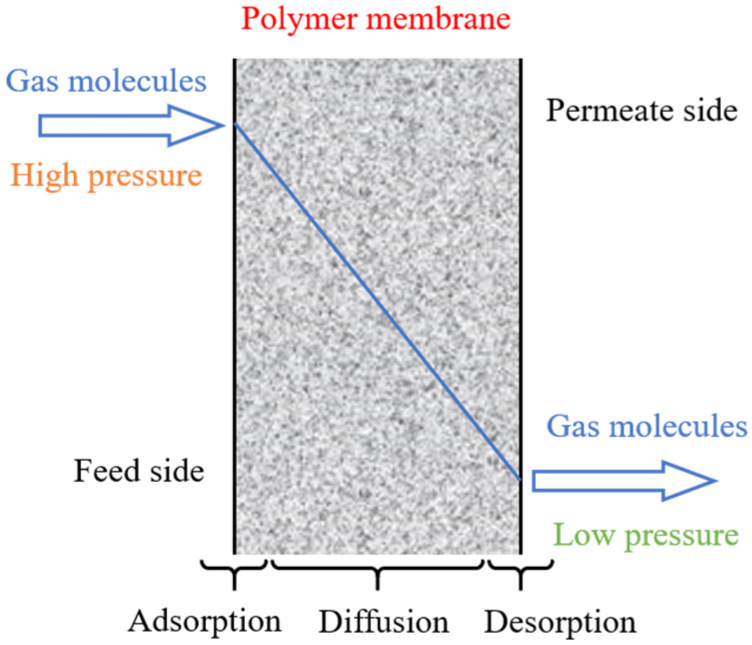
A schematic diagram of the solution-diffusion process.

As illustrated in Figure 5, according to the solution-diffusion mechanism, the gas passes through the dense membrane as follows: (1) the adsorption process: recurrence to molecular interaction, gas adsorption and dissolution occur on the membrane surface (the feed side), (2) the diffusion process: driven by the pressure, concentration or potential difference, the dissolved molecules move in the membrane layer and (3) the desorption process: the desorption of gas molecules occurs on the other membrane surface (the permeate side).

**Figure 6:**
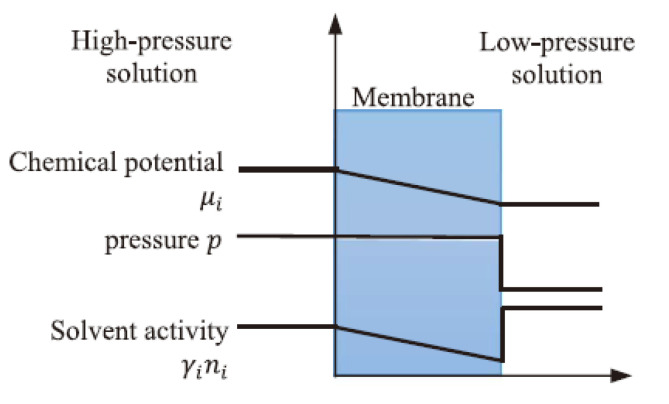
The pressure variation in the gas permeation process [41].

In the solution-diffusion process, as shown in Figure 6, the pressure within a membrane layer is uniform and drops sharply at the permeable interface of the membrane [41]. Generally speaking, the gas adsorption and desorption on the membrane surface can quickly reach an equilibrium state, while the gas diffusion process in the membrane layer is relatively slow. The gas diffusion flux at a steady state is calculated by Fick’s law:

(3) $$J=\frac{p_{1}-p_{2}}{\delta }Pt$$

(4) P=D(c)⋅S(c)

where *δ* is the membrane thickness (nm); *p* is the pressure (Pa); P is the permeability coefficient (Barrer); *t* is the travel time of molecule through the membrane (s); *c* is the concentration (mol/m^3^); *D* denotes the diffusion coefficient (cm^2^/s); *S* denotes the solution coefficient (cm^3^ (STP)/(cm^3^·cmHg)).

## 3. The Relationship between Membrane Properties and Membrane Morphology

The material properties of polymer membranes largely determine the permeable separation performance and energy efficiency of membrane gas separation technology. The dynamic properties of the polymer, the membrane pore structure and the interaction between the gas molecules and polymer membrane significantly influence the diffusion and sorption of gas molecules [42]. The plasticization of polymers also significantly affects the physicochemical properties of polymers, involving the change of molecular chain mobility and crystallinity [43,44,45]. However, it remains a challenge to obtain the afore-mentioned parameters by virtue of the existing experimental characterization and measurement methods. In view of this problem, many researchers have used molecular simulations to investigate the physical and chemical properties of polymer membranes and the main factors that dominate the adsorption and diffusion of gas molecules.

### 3.1. The Molecular Dynamics Method

**Figure 7:**
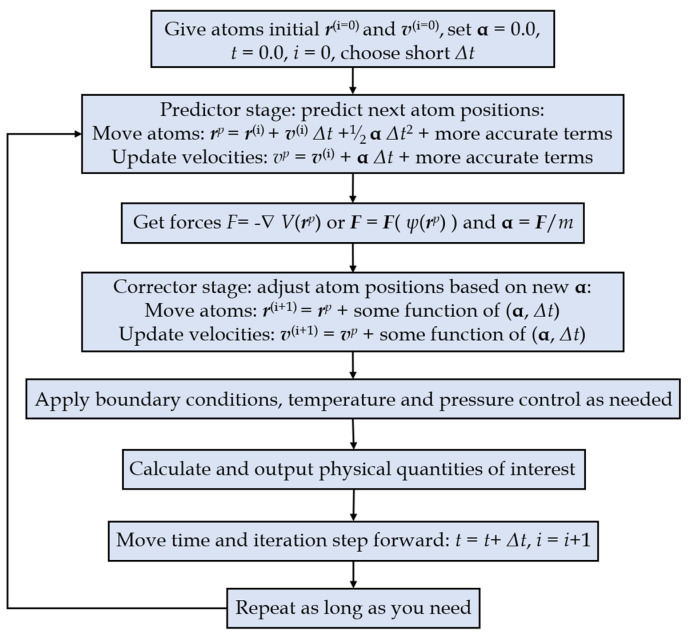
The flowchart of the MD method [46].

Molecular dynamics (MD) mainly relies on Newtonian mechanics to simulate the motion of molecular systems. Taking samples from different states of the molecular system, the integral configuration of the system is calculated and the thermodynamic quantities and other macroscopic properties of the system are obtained. The MD flow chart is depicted below (Figure 7).

Generally, the nanoscale phenomena and micromorphology are investigated with a combination of the molecular dynamics method and the Monte Carlo method in practical applications. Based on the given molecular potential energy functions and certain ensembles, Newton’s equations of motion are solved to obtain the space trajectory of each particle, namely, the microscopic state of particles changing with time. The microstructural characteristics and the macroscopic physical properties of the molecular system are further calculated according to statistical thermodynamics and physics principles [47].

### 3.2. Physical and Chemical Properties

#### 3.2.1. Densities

**Figure 8:**
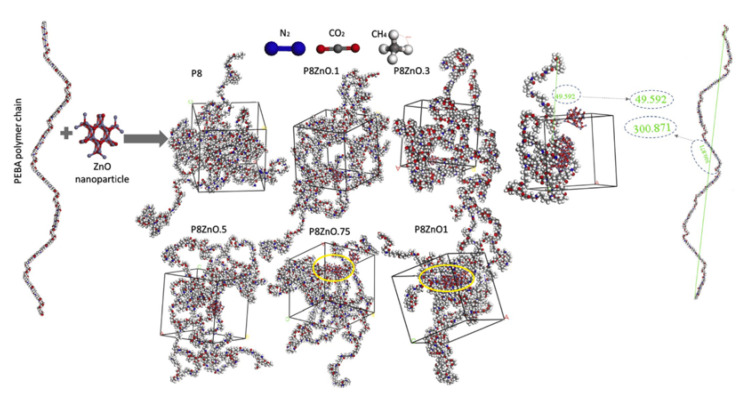
The construct procedure of PEBA chains and membranes [56].

**Figure 9:**
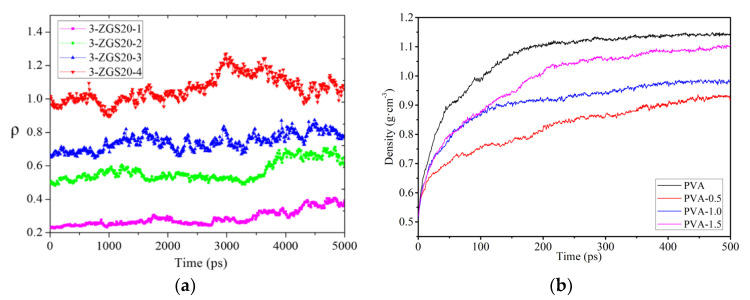
(**a**) The change in polymer density over time [54]; (**b**) the density profiles of membranes with nanoparticle loading [55].

In the construction and optimization process of molecular models, the molecular structure will gradually change and finally reach the steady state with minimum energy, as displayed in Figure 8. In general, the polymer densities at the equilibrated state of the membrane model are commonly calculated and compared with the experimental results for the validation of molecular models and simulation methods [48,49,50,51,52]. If the steady-state densities and the experimental results match well, the constructed molecular models can be guaranteed as rational and accurate and are further used to explore the polymer’s properties. In addition, the changes in polymer density also reflect the changes in the stability and compactness of the composite membrane structure [53,54,55]. For example, in Figure 9a, the increase in the number of polymer chains improves the intensity of density. The density increases slowly over time and gradually reaches an equilibrium state [54]. This suggests that the concentration of flexible poly-ether block amide (PEBA) chains increases over time near the membrane. In Figure 9b, the embedding of the nanoparticle GO might destroy the regularity of the PVA polymer chain arrangement, leading to more gaps between polymer chains and reducing the crystallinity of the nanocomposite membranes.

**Figure 10:**
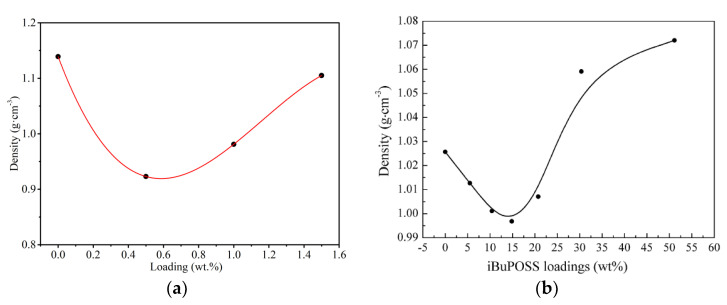
The correlation between the nanocomposite membrane density and the nanoparticle loading: (**a**) Polyvinyl Alcohol (PVA)–Graphene oxide (GO) [55]; (**b**) Poly(methyl methacrylate) (PMMA)–isobutyl (iBuPOSS) [57].

For the nanocomposite membranes, the incorporation of the nanoparticle generally causes the nonlinear variation of membrane density with the nanoparticle loading. The relationship between the nanoparticle incorporation and the polymer density can provide the inspiration for the design of nanocomposite membranes. According to the fitting curve displayed in Figure 10a, a minimum value exists for the density of the Polyvinyl Alcohol (PVA)–Graphene oxide (GO) membrane within the GO concentration range studied [55]. At large GO loads, the agglomeration of GO nanoparticles occurs in the PVA membrane, leading to an increase in membrane density. Similarly, the density of Poly(methyl methacrylate) (PMMA)–isobutyl (iBuPOSS) composites is significantly lower than that of the original PMMA at low iBuPOSS loading, but increases rapidly when the iBuPOSS loading exceeds a 15 wt.% (Figure 10b) [57]. This is attributed to the addition of the iBuPOSS molecules inducing the PMMA plasticization, resulting in a more compact arrangement of PMMA chains and a decrease in polymer density. Similar to the results in Figure 10a, iBuPOSS nanoparticles aggregate into the PMMA membrane at the iBuPOSS loading over a 15 wt.%, thus the density of the PMMA–iBuPOSS composites increases.

#### 3.2.2. The Glass Transition Temperature (*T*_g_)

**Figure 11:**
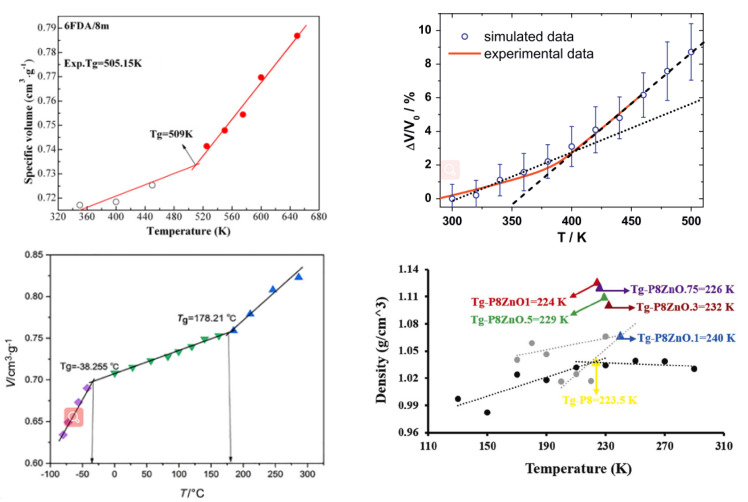
Specific volume versus temperature for composite membranes [51,52,56,62].

The flexibility of polymer chains significantly affects the diffusion properties of small molecules in polymer membranes, which is usually characterized by the glass transition temperature (*T*_g_). The procedure for calculating *T*_g_ in the molecular simulation has been explained in detail in many publications [48,58,59]. *T*_g_ can be obtained at the point where the two slopes (the fitting curve of specific volume versus temperature) intersect, as shown in Figure 11. Two important factors influencing *T*_g_ are the strength of the chains’ interaction and the chain flexibility [60]. The number and size of substituents influence the rotatability of the polymer chain [61]. In general, the higher the *T*_g_ value, the weaker the migration ability of the molecular chains.

#### 3.2.3. Fractional Free Volume (FFV)

**Figure 12:**
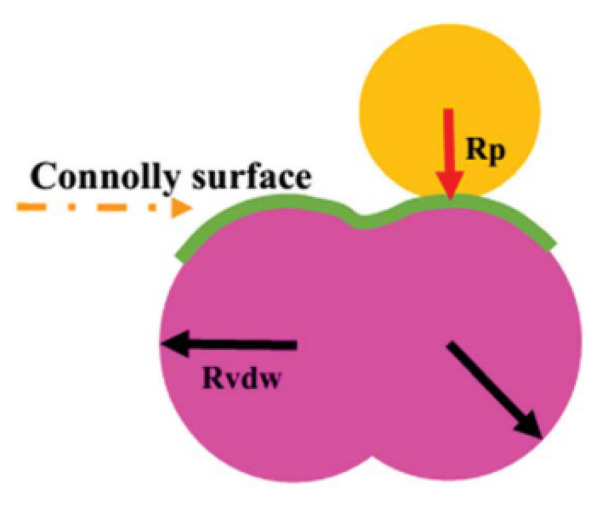
A schematic diagram for calculating free volume [67].

**Figure 13:**
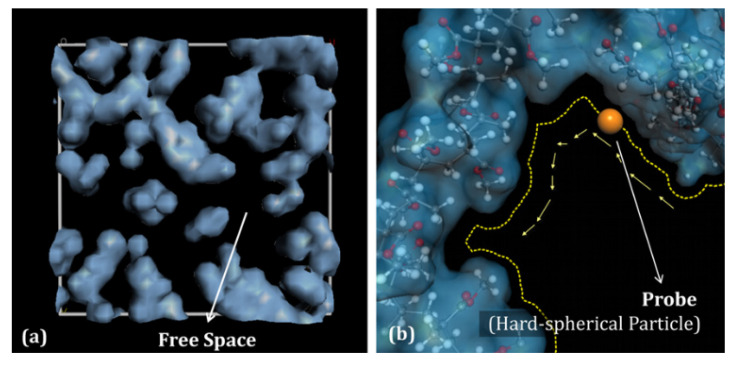
(**a**) Free space and (**b**) van der Waals volume in a molecular model [50].

The transport behaviors of small gas molecules in polymer membranes are greatly affected by the free volume and its distribution [50,51,63,64]. As demonstrated in Figure 12, the free volume of the equilibrated polymer membranes is commonly discussed by the feat of the “Connolly surface” method [65]. In Figure 13, the free space and accessible volume in the membrane model are marked and indicated. The atomic boundary of the membrane is reconstructed by the hard spheres with van der Waals radius. The *FFV* of the polymer membrane is measured by a hard spherical probe. As a hard-spherical particle rolls over the van der Waals surface, the Connolly surface is labeled. The free volume can be calculated according to the van der Waals volume (*V_VdW_*) divided by the Connolly surface. Generally, *FFV* is defined as follows:

(5) $$FFV=\frac{\left(V_{s}-1.3V_{VdW}\right)}{V_{s}}$$

where *V_s_* is the specific volume, namely, the reciprocal density [66].

**Figure 14:**
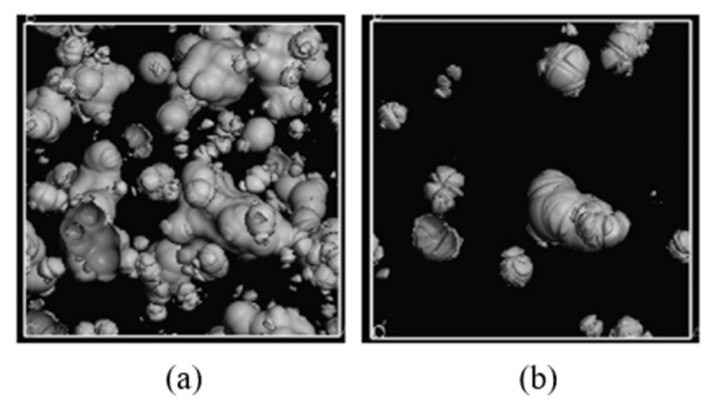
The free volume holes in PVA–EDTMPA-20 with water and propylene as the probes: (**a**) water; (**b**) propylene [68].

The size and morphology of the free volume play an important role in the diffusion behavior of small molecules in the polymer. The free volume provides the necessary activity space for the chain motion and the diffusion space of small molecules. In general, a lower *FFV* indicates fewer free volume holes in the polymer membrane, thus the distance required for small molecules to jump between free volume holes in the membrane will be increased. Correspondingly, the diffusion probability of small molecules in the polymer membrane might reduce. The unreasonable distribution of free volume holes is also not conducive to mass transfer behavior in the polymer membrane [67]. As shown in Figure 14, the free volume holes with water and propylene as the probes were investigated by Pan et al. [68]. In the PVA–EDTMPA membrane, the free volume holes with water as the probe are almost connected with each other, while the holes with propylene as the probe are dispersed sparsely. The better the connectivity of free volume holes, the easier the diffusion of the gas molecules; therefore, the water molecules possess a better diffusivity in the PVA–EDTMPA membrane than the propylene molecules.

In the existing research, density, temperature and the free volume fraction are commonly used to characterize the membrane morphology and preliminarily predict the correlation between the membrane microstructure changes and the membrane diffusion-separation properties. However, future research should focus on the following aspects: (1) the rationality and accuracy of constructed molecular models need to be compared and verified by more physical and chemical properties of membrane materials, such as molecular spectra or osmotic separation parameters and (2) the prediction of the correlation between the diffusion, the adsorption properties of gas molecules and the microscopic properties of polymer membranes is mostly based on theoretical analysis, which needs to be supported by more experimental data. It is also necessary to develop and establish a more refined and credible prediction method.

### 3.3. Interfacial Interactions

**Figure 15:**
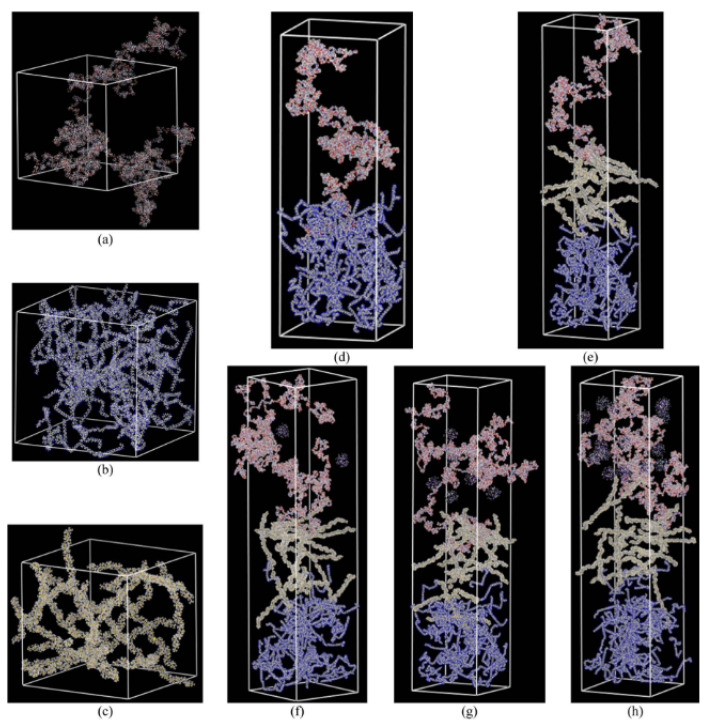
The final molecular configuration of amorphous cells of pristine membranes and nanocomposite polymeric membranes: (**a**) Pebax, (**b**) PAN and (**c**) PTMSP; composite polymeric membranes: (**d**) CPM2 and (**e**) CPM3 and nanocomposite polymeric membranes (**f**) NCPM3/ZIF8%, (**g**) NCPM3/ZIF22% and (**h**) NCPM3/ZIF34% [31].

As demonstrated in Figure 15, the interlayer compatibility and complementarity of the composite membranes are important for the diffusion properties of gas molecules. Based on the current membrane fabrication process, three approaches to improve the structural stability of composite membranes include: the cross-linking modification of the separation layer [69,70], the multilayer membrane structure [71,72] and the copolymer membrane structure [73,74]. Based on the constructed multilayer molecular model, the interfacial compatibility can be analyzed comprehensively by the interfacial energies and the polymer solubility parameters.

The interfacial energies of the composite membrane (*E*_int_) can be calculated by the difference between the potential energy of the multilayer structural membrane (*E*_layer,1-2_) and that of the single-layer polymer (*E*_layer,1_, *E*_layer,2_) [75,76]:

(6) $$E_{\mathrm{int}}=E_{\mathrm{layer},1-2}-(E_{\mathrm{layer},\;1}+E_{\mathrm{layer},\;2})$$

where *E*_layer,1-2_ is the potential energy of the composite membrane. *E*_layer,1_ and *E*_layer,2_ are the potential energies of the single-layer polymers, respectively. A negative value of *E*_int_ indicates that there is gravity between the support membrane and the selective layer. In addition, the greater the absolute value of *E*_int_, the stronger the interaction between two membrane layers.

**Figure 16:**
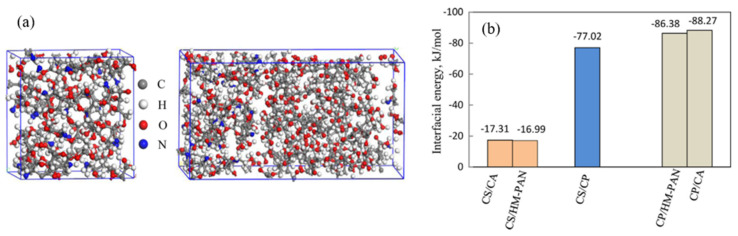
(**a**) The molecular models of the CS active layer and the CS/CP interfacial membrane; (**b**)the interfacial energy of the CS/CP composite membrane [77].

**Figure 17:**
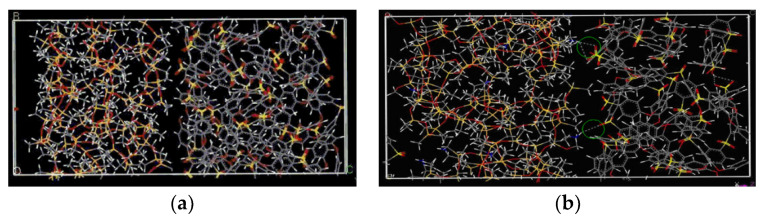
(**a**) The model for the PDMS–TEOS-0.06/PES composite membrane; (**b**) the model for the PDMS–APTMS-0.06/PES composite membrane [78].

Wang et al. [77] investigated the interfacial energies and the interfacial compatibility between the chitosan (CS) selective layer, the bioadhesive carbopol (CP) interlayer and the polysulfone (PS) support membrane (Figure 16a). The results in Figure 16b indicate that the interfacial interaction of CS/CA and CS/HM–PAN composite membranes is enhanced by the introduction of the CP intermediate layer, thus improving the structural stability. In order to improve the interfacial stability between the polydimethylsiloxane (PDMS) separation layer and the polyether sulfone (PES) porous membrane, Wu et al. [78] added a bifunctional aminosilane, *γ*-Aminopropyltrimethoxysilane (APTMS), into the active layer, as shown in Figure 17. The results show that no intermolecular hydrogen bond is formed at the interface of the PDMS–TEOS-0.06 membrane and the PES membrane, indicating that APTMS instead of TEOS is beneficial to improve the interface interaction. The introduction of polar APTMS enhances the hydrophilicity of the PDMS layer and thus improves the interface compatibility.

**Figure 18:**
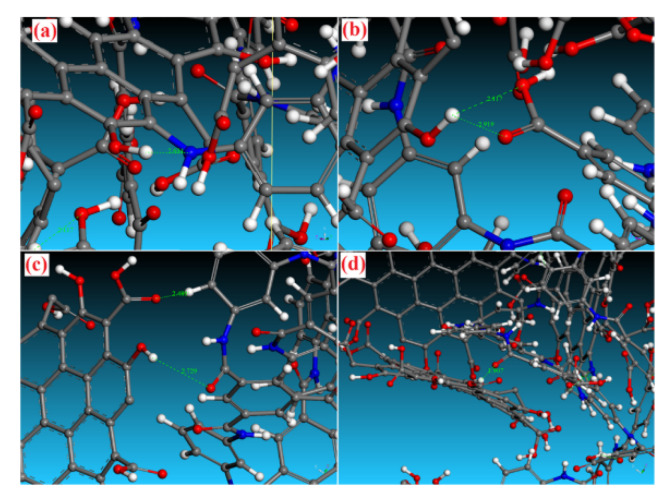
The formation of H-bonds between the GQDs and the PA chain functional groups [35].

The interfacial energy of the composite membrane arises from non-bonded interactions, including the hydrogen bond, electrostatic force and van der Waals force [31,62]. Salestan et al. [35] reported that the interface compatibility of PA chains and the GQDs in the nanocomposite membrane was enhanced due to the formation of covalent bonds and hydrogen bonds. As depicted in Figure 18, the minimum acceptable angle and the maximum acceptable distance of hydrogen bonds were set to 90° and 2.9 Å, respectively, in their H-bond detection study, which verified the presence of hydrogen bonds in the structures based on Jeffrey’s categorization [79]. Due to the presence of donor and acceptor groups in the structure of both the GQDs and the PA, a large number of hydrogen bonds are formed between the GQDs and the PA chain functional groups. Zhao et al. [80] found that a strong attraction existed at the interface between polyacrylonitrile (PAN) and gelatin (GE), in which the van der Waals force was dominant. The solubility parameters, mean-square displacement and interface energies suggested that there is a moderate interface compatibility between the selective layer and the support membrane.

The above literature shows that the energy analysis has important guiding significance for the material selection and the fabrication of the composite membrane, the copolymer membrane and the nanoparticle filled membrane. However, future research should focus on the following aspects. (1) Although the molecular force field has been widely used in molecular dynamics, the studies of energy analysis based on molecular force field are not sufficiently in-depth. The analysis of energy decomposition in molecular simulation should be combined with molecular chemistry theories to deliver reasonable and instructive conclusions. (2) The interaction and entanglement between the polymers may show that they possess a particular molecular morphology. Each molecule or atom in the polymer will be affected by its surrounding molecules and will react differently under different temperatures, pressures and strains. The effects of various operative conditions should be investigated and considered in the construction of molecular models and the properties investigated. (3) Due to the simple functional form of the molecular force field, the accuracy of the energy decomposition using the molecular simulation method is still far from that of the wave function-based method; therefore, the accuracy of the quantitative results is poor. The potential functions of the molecular force field are rarely addressed or discussed by researchers and this may need to be improved for specific molecular configurations in the future.

## 4. Transport Characteristics

The factors affecting the transport efficiency in the gas transportation process mainly include solubility, diffusion, the permeability coefficients and the selectivity of the polymer membranes. These transport characteristics are closely related to the physical and chemical properties and microstructure of the separation membranes.

### 4.1. Adsorption

From the perspective of thermodynamics, the gas adsorption in polymers involves the condensation of the gases and the mixing of molecules and polymers. The solubility coefficient (*S*) is dominated by the gas condensability and the interaction between the polymer and the gas molecules. The condensability can be determined by the gas critical temperature. In general, the solubility coefficient (*S*) increases with the gas condensation ability. The interaction between the polymer and the gas molecules can be analyzed using the Monte Carlo (MC) simulation.

The MC simulation is a calculation method based on probability and statistical theory. It uses random numbers to realize a statistical simulation or sampling to obtain approximate solutions to complex problems [81]. Numerous studies [31,78,82,83] have introduced the computational logic and procedures of the MC simulation method. The solubility coefficient is calculated by using the GCMC method according to the change curve of the adsorbed molecule concentration with pressure, which is generally defined as the limit slope of the curve at zero pressure:

(7) $$S=\underset{p\to 0}{\lim}\left(\frac{c}{p}\right)$$

where *c* is the concentration of the absorbed molecule in the membrane, mol/m^3^.

**Figure 19:**
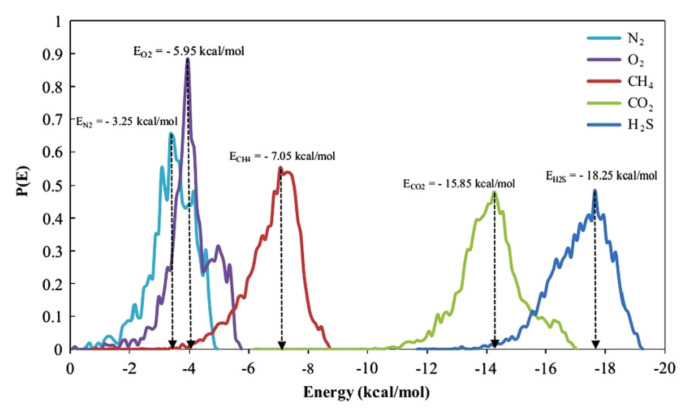
The adsorption energy distribution of CO_2_, CH_4_, O_2_, N_2_ and H_2_S gas molecules (*P* = 0~10 bar, *T* = 298 K) [48].

Amani et al. [48] examined the solubility of poly(urethane–urea)s (PUUs) membrane for various gas molecules. The order of the solubility obtained is consistent with that of the gas condensability, which is H_2_S > CO_2_ > CH_4_ > O_2_ > N_2_. Since H_2_S and CO_2_ are polar gases, the solubility of H_2_S and CO_2_ is stronger than that of other gases. Five peaks at −15.85, −7.05, −5.95, −3.25 and −18.25 kcal/mol, respectively, can be observed in Figure 19 for CO_2_, CH_4_, O_2_, N_2_ and H_2_S gases. The high interaction energy of H_2_S with the PUUs membrane indicates a strong affinity of the PUUs membrane with H_2_S, so H_2_S has a high solubility in the PUUs membrane. The solubility of CH_4_, N_2_ and O_2_ in the PUUs membrane is similar, but is less than that of CO_2_ and H_2_S.

**Figure 20:**
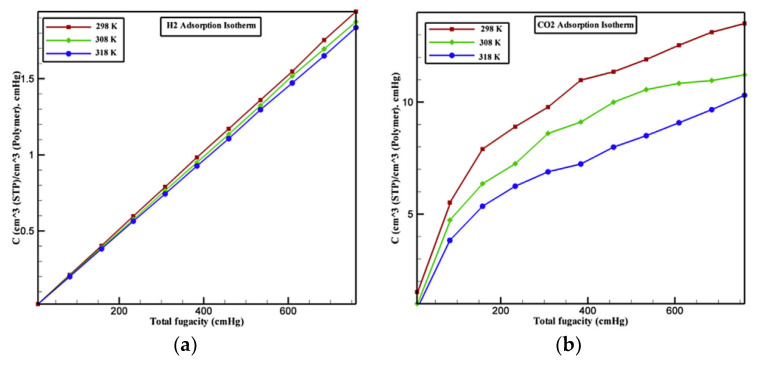

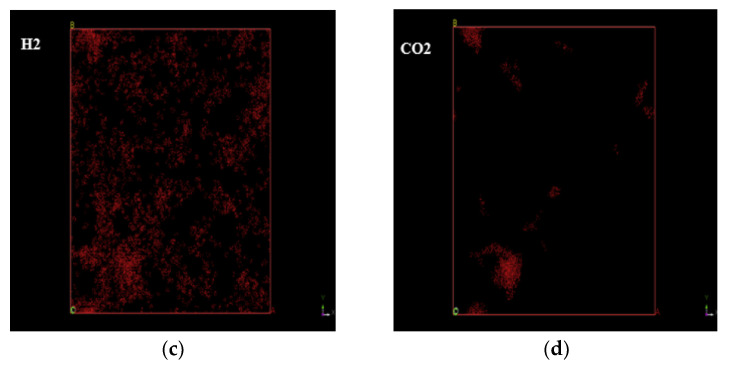
The isotherm adsorption curves of CO_2_ (**a**) and H_2_ (**b**) in the polymer membrane; the adsorption sites of H_2_ (**c**) and CO_2_ (**d**) in polyurethane [49].

In the polymeric matrix, the two key aspects of the molecular adsorption sites are the free volume voids and the spaces between molecular chains [49]. At low pressures, gas molecules are usually trapped by the free volume holes in the polymer membrane, following the Langmuir and Henry’s sorption law. Meanwhile, gas molecules that are not captured enter the spaces between the molecular chains. At high pressures, the free volume holes are mostly occupied by gas molecules, so the Langmuir adsorption is no longer the dominant sorption mechanism, but Henry’s sorption law becomes dominant. As displayed in Figure 20a, the isothermal curve shows a linear upward trend, implying that the dominant sorption mechanism is Henry’s sorption law for H_2_. In view of CO_2_, the dual-mode sorption mechanism dominates its adsorption process. The gas adsorption capacity gradually reaches saturation for the two gases at a high pressure, which indicates the additional effect of Henry’s sorption. Figure 20b clearly shows CO_2_ and H_2_ sorption sites in the polymeric membrane. Due to its small size and strong activity, the H_2_ molecule has more opportunity to access the free volume holes and the spaces between molecular chains compared with the CO_2_ molecule.

**Figure 21:**
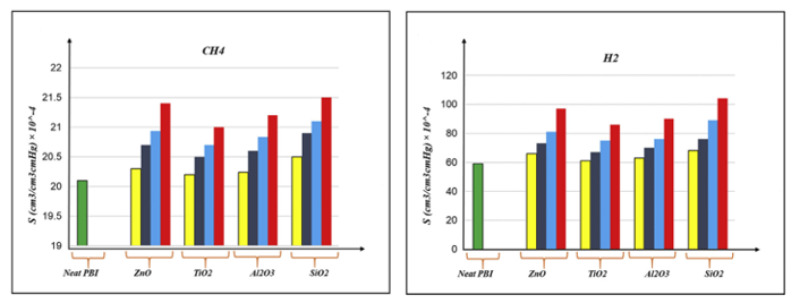
Solubility of H_2_ and CH_4_ in membranes filled with different nano-oxide materials (*P* = 4 atm, T = 298 K) [53].

**Figure 22:**
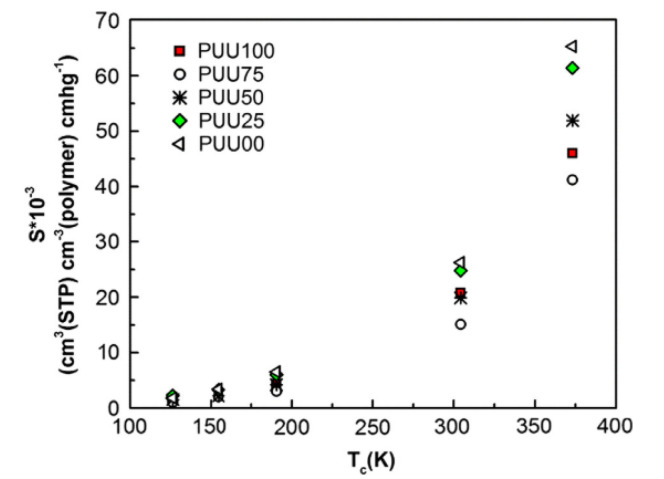
The relationship between solubility and critical temperature of the gases [48].

Khosravanian et al. [53] calculated the solubility coefficients of H_2-_ and CH_4-_penetrated molecules in the poly(benzimidazoles)/nanoparticle oxides’ composites. As shown in Figure 21, the addition on the nano-oxide materials enhanced the H_2_ solubility, but weakened the CH_4_ solubility. The results indicate that the nanomaterial type is significant for the solubility, gas transport characteristics and H_2_ capture in the polymer membranes. Amani et al. [48] investigated the relationship between the solubility of gas molecules and the critical temperature (Figure 22). The results showed that the solubility coefficient increased with the critical temperature.

The GCMC simulation method effectively characterizes the kinetic mechanism of the polymer membrane adsorbing other substances from smaller spatial and temporal scales. Based on the above literature analysis, the adsorption energy, the adsorption site and the variation curve of molecule adsorption concentration can be used to comprehensively analyze the difference in the adsorption properties of polymer membranes for various gas molecules. Combined with the quantitative calculation of the solubility coefficient, the solubility properties of polymer membranes can be effectively predicted. Nevertheless, it is necessary to carry out in-depth research in the following aspects. (1) In the study of the gas-membrane adsorption mechanism, the key to the MC method is to establish the micropores’ structural model of membrane materials and the interaction model between the gas molecules and the membrane materials. Further research is needed to simplify appropriate adsorption models from complex practical problems. (2) The adsorption effect of polymer membranes is affected by the pore size, temperature, pressure, environment (such as humidity) and other important parameters. For a variety of factors, the selection of suitable operating conditions to obtain a relatively balanced performance is also an important direction of research.

### 4.2. Diffusion

The diffusivity of gas molecules in membranes is strongly affected by the free volume and the mobility of polymer chains [31,49,62,84,85,86,87]. The connectivity, distribution and size of free volume cavities are significant for gas molecule diffusion behavior [55,88]. The local mobility of molecular chains affects the diffusion coefficient, which is manifested as a change of diffusion selectivity. However, it is difficult to quantify the correlation between physicochemical properties and the diffusion coefficient. A deep understanding of the relationship between the physicochemical properties of membranes and diffusion is necessary and is conducive to obtaining the correlation data through the analysis of the free volume fraction, void size and local molecular chain motion.

**Figure 23:**
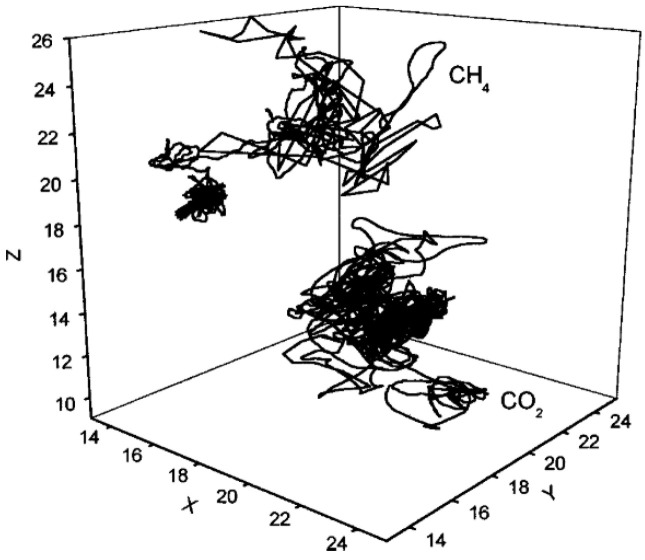
The local trajectories of CH_4_ and CO_2_ in the polyetherimide [89].

Figure 23 presents the typical trajectories of CH_4_ and CO_2_ in the polyetherimide (PEI) matrix [89] and clearly indicates the gas molecules oscillating within the free volume holes and jumping between holes. The diffusion behavior of the gas molecules actually consists of random local oscillations inside the microcavities and occasional jumps between two microcavities. The oscillations amplitude is highly relevant to the size of the accessible holes. The longer the gas molecules stay in the microcavity, the less the jump between the microcavities, which indicates that the gas molecules have a small self-diffusion coefficient. As evident from the gas displacements (Figure 23), the CH_4_ molecules had a faster motion compared with the CO_2_ molecules. Thus, it can be predicted that CH_4_ has a greater self-diffusion coefficient.

**Figure 24:**
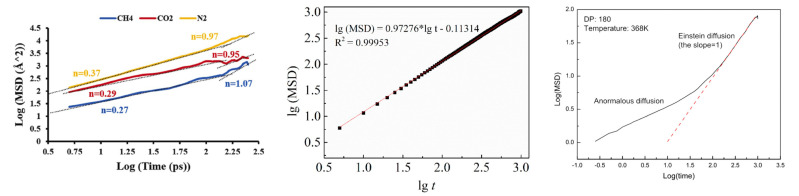
The mean square displacement of gas molecules in the membrane matrix [56,84,90].

The mean square displacement (MSD) of gas molecules typically includes collisions inside the free volume and the jump between the adjacent free volume holes. As shown in Figure 24, the result of repeated jumps is the diffuse motion of the gas molecule characterized by a linear MSD in time. Using Molecular Dynamic (MD) simulation, the increase in the MSD with time is related to the diffusion coefficient, *D*:

(8) $$D_{i}=\frac{1}{6N_{\alpha }}\underset{t\to \infty }{\lim}\frac{d}{dt}\sum_{i=1}^{N_{\alpha }}\langle {\left[r_{i}(t)-r_{i}(0)\right]}^{2}\rangle$$

where *N*_α_ is the number of diffusive atoms in the system. Usually, judging whether the diffusion is within the normal range depends on the logarithmic plot of the MSD. When the curve slope is close to 1, the diffusion falls within the normal range, which applies to Einstein’s formula.

**Figure 25:**
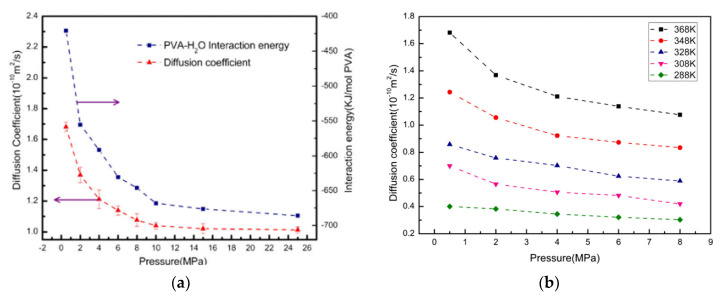
(**a**) Diffusion coefficients and the interaction energy of PVA–H_2_O versus pressures (T = 368 K); (**b**) diffusion coefficients of H_2_O versus pressure in PVA at different temperatures [90].

Fei et al. [90] calculated the diffusion coefficient of H_2_O molecules in a poly(vinyl alcohol) membrane. As seen in Figure 25, the increase in the interaction energy with the pressure impedes the diffusion of water molecules, thus the diffusion coefficients of H_2_O molecules decrease. Under a pressure over 10 MPa, the number of H_2_O molecules captured by the PVA chains reaches saturation and the increasing trend of the intermolecular force between H_2_O molecules and PVA chains becomes weak. As shown in Figure 25b, the diffusion coefficient at different temperatures shows a downward trend with the increase in pressure, due to the augmentation of the intermolecular force of PVA–H_2_O.

The molecular trajectories and the MSD change curves are conducive to judging the motion form and jump amplitude of small molecules in the porous membrane. The quantitative calculation results of the diffusion coefficient and molecular trajectory confirm each other. These results can comprehensively analyze the factors affecting the molecular diffusion coefficient. Nevertheless, the following research issues are important to investigate in the future. (1) The study of the diffusion process of pure components cannot comprehensively investigate the diffusion properties of composite membranes. An investigation into the diffusion behavior of binary or multiple mixed components is closer to the actual situation. The research on the diffusion of mixed components has more significance for the separation and chemical reactions of mixtures. (2) The self-diffusion coefficient determined by the molecular dynamics simulations is accurate in a certain range and agrees with the diffusion coefficient measured by an experimental method. However, due to the existence of the “slow” diffusion phenomenon in the process of a dynamics simulation, the diffusion coefficient of molecules with a large kinetic diameter measured by the molecular dynamics method is not accurate and the development of an advanced approach for solving this problem is challenging work. (3) For the molecular dynamics simulation, the key problem is to provide the accurate interaction potential energy between the molecules or atoms. The empirical potential energy parameters cannot meet the needs of some simulations. The calculation of the interaction energy between particles through quantum chemistry may be an effective method to obtain the potential energy parameters and is an important direction of future development.

### 4.3. Permeability and Selectivity

**Figure 26:**
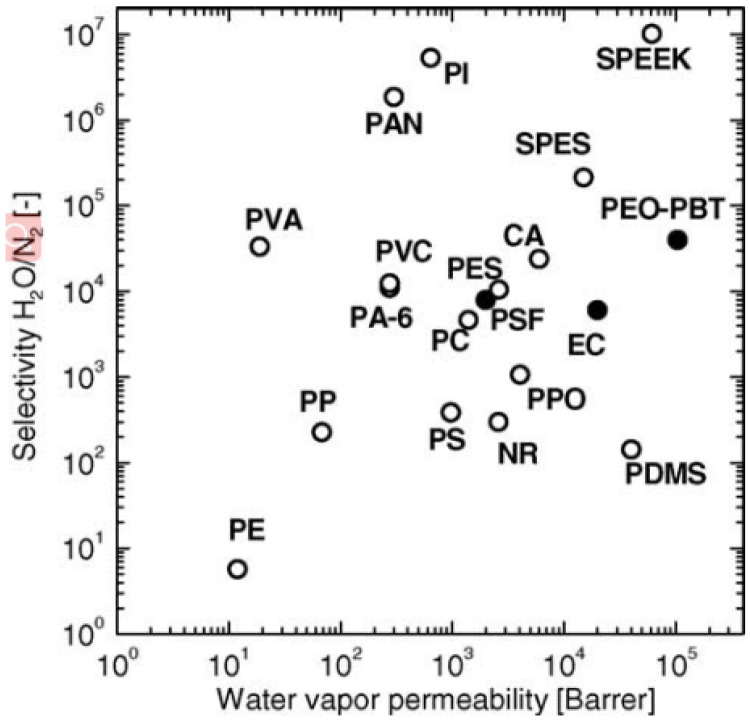
The permeability and selectivity of water vapor and nitrogen in various polymers at 30 °C [91].

**Figure 27:**
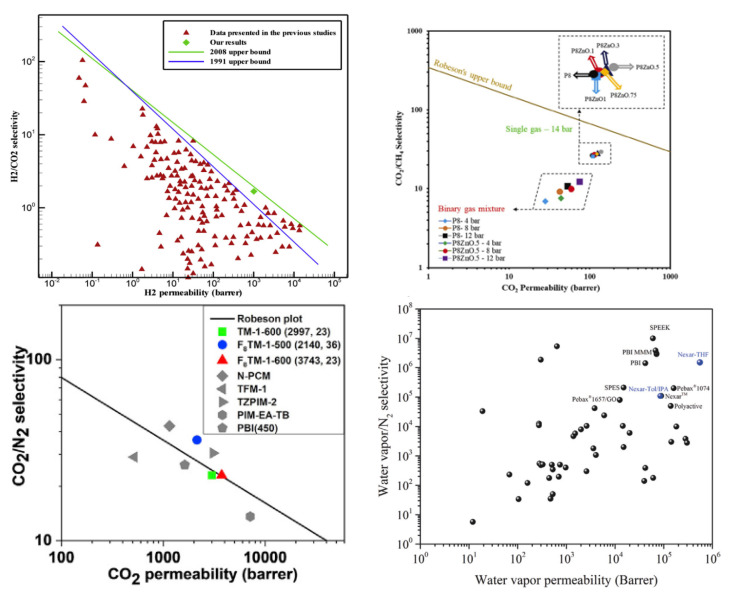
Robeson’s upper bound of various gases in different membranes [49,56,93,94].

Figure 26 summarizes the permeability and selectivity of water vapor and nitrogen in various polymers at 30 °C. The trade-off between selectivity and permeability is a common phenomenon for most polymer membrane materials [91]. The Robeson upper bound plot reflects the separation performance limit of the homogeneous polymer membrane for a specific gas, which is conducive to guiding the optimization and breakthrough of the polymer membrane structure/performance [92,93]. The two pivotal parameters in the Robeson upper bound plot are the permeability coefficient (P) and the separation factor (*α*) of the component to be separated. The permeability coefficient indicates the speed at which the gas molecules transport through the membrane, while the separation factors indicate the separation degree of the target molecules from other molecules. Figure 27 displays the Robeson’s upper bound of different binary gas pairs in the polymer membranes [49,56,93,94]. The closer that the data point of the membrane is to the upper bound line, the better the gas separation performance of the polymer membrane. These fabricated membranes are attractive and suitable to be applied in industrial applications and they are also predicted to have low initial investment and operating costs. If the data points exceed the Robeson’s upper bound, the membrane could be considered as a candidate for a commercial membrane. In future research, more modification methods of the membrane material should be explored to break though the Robeson’s upper bound and promote the polymer membranes’ performance. Referring to the molecular simulation method, the novel microstructures of the membrane polymer (such as the copolymer membrane, the nanocomposite membrane and the organic–inorganic composite membrane) can be constructed to explore and predict the construction scheme of the membrane material with a better separation performance.

## 5. Conclusions and Perspectives

The exploration on the morphology and material properties of the gas separation membrane by molecular simulation has been reviewed in this paper. Compared with the traditional experimental methods, molecular simulation can explore the microscopic behavior of small molecules and polymer chains at the atomic or molecular level. By calculating the physical and chemical properties of the polymer membrane and the adsorption and diffusion behavior of small molecules in the membrane, the internal relationship between the material properties and separation performance of the gas separation membrane can be clarified. The relevant research results can provide a theoretical basis for the design and application of polymer membrane materials.

However, the previous studies on the application of molecular simulation in gas membrane separation still have several deficiencies:

(1) A large number of studies focus on the solubility and diffusion coefficients of polymer membranes, and few studies report the relationship between membrane morphology, properties and membrane permeability and selectivity;
(2) In most calculations, gas molecules are simplified as rigid molecules. The potential energy equations of gas–gas and gas–membrane interactions generally only consider L-J potential energy and coulomb force. The collision between molecules and walls is considered as elastic collision. Based on the above assumptions, the molecular simulation is still far from an accurate quantitative analysis of the actual membrane separation process;
(3) The existing empirical potential energy parameters cannot meet the needs of some simulations. The necessary potential energy parameters and suitable force field equations are lacking. Considerable work should be undertaken in parameter selection and molecular model building;
(4) At present, there are few commercial products of composite polymer membranes used for gas separation. The parameters required for modeling (the material properties of the separation layer, crystal type and microscopic shape of membrane pore, etc.) and the experimental data for comparing simulation results are scarce.

Therefore, from the perspective of membrane materials’ design, there still are many problems to be studied for the gas membrane separation process by using molecular simulation technology. It is only through collective investigations on improving above-mentioned aspects that membrane processes can continue to innovate and push the boundaries of membrane separation technology. Meanwhile, some suggestions are put forward for further exploration: (1) the rationality and accuracy of constructed molecular models need to be compared and verified by more experimental data related to the physicochemical properties of membrane materials, (2) the construction and optimization of the multilayer molecular models need further research, to explore and predict the gas transport characteristics in the composite membranes and (3) for the design of gas separation membrane materials, molecular simulation methods and experimental methods should complement each other in future research, to comprehensively determine the most appropriate membrane material construction scheme for the micro and macro levels.

## Data Availability

Not applicable.

## Nomenclature

*c*	Concentration (mol/m^3^)	P	The permeability coefficient (Barrer)
*D*	Diffusion coefficient (cm^2^/s)	*p*	The pressure (Pa)
*D_s_*	Surface diffusion coefficient (cm^2^/s)	*R*	The gas constant (J/mol K)
*d*_1_/*d*_2_	The membrane thickness changes due to the surface adsorption	*r_p_*	The membrane pore radius (m)
*E*_int_	The interfacial energies of the composite membrane (kcal/mol)	*S*	The solution coefficient (cm^3^ (STP)/(cm^3^ kPa))
*E*_layer,1-2_	The total energies of the composite membrane (kcal/mol)	*T*	The temperature (K)
*E*_layer,1_	The energies of separation layer (kcal/mol)	*V*_s_	The reciprocal density
*E*_layer,2_	The energies of support layer (kcal/mol)	*V*_VdW_	The van der Waals volume
*FFV*	Fractional free volume (%)	*Greek*	
*f_k_*	The gas flux due to Knudsen diffusion (kg/(m^2^ s))	*ρ*	The density (kg/m^3^)
*f_s_*	The gas flux due to surface diffusion (kg/(m^2^ s))	*ε*	The porosity of porous media
*M*	The gas molecular weight (kg/mol)	*μ_s_*	The shape factor
*N*_α_	The atoms number	α	The selectivity
